# Supplementary material for: Early Parenteral Administration of Ceftiofur has Gender-Specific Short- and Long-Term Effects on the Fecal Microbiota and Growth in Pigs from the Suckling to Growing Phase
Source: Animals (Basel). 2019 Dec 20;10(1):17. doi: 10.3390/ani10010017 (PMC7022567; doi:10.3390/ani10010017)
Supplement: Supplementary file 1 [file animals-10-00017-s001.pdf]

Supplementary Table 1. Average daily weight gain (ADWG) in gram (g) in female (♀) and male (♂) pigs receiving either the antibiotic (AB) or control (Control) treatment between time periods days 0-12, day 0-28, day 0-97, day 12-28, day 28-75, day 28-97, day 75-97.

	ADWG g				
	♀		♂		
Time period	Control	AB	Control	AB	SEM
day 0-12*	172	166	171	170	6.7
day 0-28 **	243	233	237	236	7.0
day 0-97*	495	478	488	507	14.8
day 12-28**	290	278	276	277	8.8
day 28-75*	499	187	475	492	15.9
day 28-97	592	579	586	617	16.6
day 75-97 †	788 $^{B\ ab}$	772 $^{AB\ b}$	817 ^{ABab}	879 ^{Aa}	31.0

Data are presented as least-square means ± pooled standard error of the mean (SEM). ^{A,B} Different capital letters indicate a tendency (P<0.1); ^{a,b} Different lowercase letters indicate significant differences (P<0.05). ** Litter effect P<0.001; * Litter effect P<0.05; † Sex effect P<0.05.

Supplementary Table 2. Permutational multivariate analysis of variance (**PERMANOVA**) results for ceftiofur effects on faecal microbiome.

Source of Variation	df	SS	R^2	F	*p*-value
Sow	1	0.48	0.011	3.137	0.029
Sex	1	0.04	0.001	0.277	0.879
Treatment	1	0.16	0.003	10.209	0.348
Day	1	16.73	0.003	110.034	0.001
Sow: Sex	1	0.04	0.001	0.251	0.896
Sow: Treatment	1	0.14	0.003	0.887	0.400
Sex: Treatment	1	0.03	0.001	0.162	0.954
Sow: Day	1	0.15	0.003	0.998	0.341
Sex: Day	1	0.03	0.001	0.216	0.941
Treatment: Day	1	0.09	0.002	0.556	0.588
Sow: Sex: Treatment	1	0.06	0.001	0.389	0.764
Sow: Sex: Day	1	0.08	0.002	0.547	0.593
Sow: Treatment: Day	1	0.08	0.002	0.513	0.656
Sex: Treatment: Day	1	0.05	0.001	0.354	0.789
Sow: Sex: Treatment: Day	1	0.06	0.001	0.377	0.775
Residual	174	26.45	0.592		
Total	189	44.65	1.000		

The analysis based on pairwise distance of a multivariate data set and values were obtained using type III sums of squares with 999 permutations of residuals, considering significant difference at $p < 0.05$. df, degrees of freedom; SS, sum of squares.

Supplementary Table 3. Relative abundance (% of total reads) of bacterial phyla in female (♀) and male (♂) pigs receiving either the antibiotic (AB) or control (Control) treatment on days 0, 12, 28 and 97 of life.

	day 0					day 12				
	♀		♂			♀		♂		
	Control	AB	Control	AB	SEM	Control	AB	Control	AB	SEM
Proteobacteria **	83.75	87.26	88.50	87.03	5.17	57.85 ^{AB}	69.02 ^{A}	52.26 ^{B}	57.45 ^{AB}	6.95
Firmicutes **	15.67	12.49	11.13	12.84	5.10	35.14 ^{AB}	21.23 ^{B}	39.34 ^{A}	29.70 ^{AB}	6.51
Actinobacteria **	0.25	0.16	0.15	0.05	0.11	1.78 ^{B}	3.60 ^{AB}	2.04 ^{AB}	5.31 ^{A}	1.42
Bacteroidetes **	0.07	0.02	0.09	0.01	1.29	0.44	0.13	1.11	0.41	1.34
Verrucomicrobia	0.01	0	0.01	0	0.50	1.89 ^{a}	0.08 ^{b}	0.14 ^{b}	0.36 ^{b}	0.52
Archaea ** †	0.09	0.02	0.05	0.01	0.33	0.32	0.34	0.52	0.66	0.35
Elusimicrobia ** ¤	0	0	0	0	0.08	0.00	0.00	0.00	0.00	0.09
Tenericutes ** ◊	0.05	0.01	0.05	0	0.09	0.09	0.01	0.16	0.16	0.09
Synergistetes **	0.02	0	0	0.01	0.86	2.15	3.30	3.97	2.79	0.89
Others	0.10	0.03	0.03	0.05	0.30	0.21	0.12	0.47	1.02	0.31

	day 28					day 97				
	♀		♂			♀		♂		
	Control	AB	Control	AB	SEM	Control	AB	Control	AB	SEM
Proteobacteria **	10.12	4.47	8.43	8.47	2.54	16.45 ^{B}	28.94 ^{A}	23.28 ^{AB}	23.02 ^{AB}	4.54
Firmicutes **	73.78	79.08	72.63	73.74	4.79	73.88	65.96	65.55	69.78	5.06
Actinobacteria **	0.66	0.46	0.41	0.49	0.20	0.48	0.45	0.46	0.52	0.06
Bacteroidetes **	8.83 ^{AB}	7.53 ^{AB}	5.74 ^{B}	10.34 ^{A}	1.83	3.47 ^{AB}	2.06 ^{B}	7.15 ^{A}	2.84 ^{B}	1.83
Verrucomicrobia	0.33	0.53	1.80	1.33	0.71	0.30	0.14	0.10	0.14	0.71
Archaea ** †	3.44 ^{AB}	2.45 ^{B}	3.65 ^{A}	2.74 ^{AB}	0.48	2.38 ^{a}	0.98 ^{b}	1.52 ^{ab}	1.32 ^{ab}	0.48
Elusimicrobia ** ¤	0.26 ^{b}	0.07 ^{b}	0.77 ^{a}	0.31 ^{b}	0.12	0.10	0.02	0.07	0.11	0.12
Tenericutes ** ◊	0.35 ^{b}	0.88 ^{a}	0.63 ^{ab}	0.42 ^{b}	0.13	2.14 ^{a}	1.42 ^{b}	1.61 ^{b}	1.89 ^{b}	0.13
Synergistetes **	1.96	2.77	3.70	1.55	1.23	0.10	0.12	0.17	0.06	1.23
Others	0.56	1.03	0.69	1.20	0.76	1.49	0.60	0.68	0.70	0.49

Data are presented as least-square means ± pooled standard error of the mean (SEM). ^{A,B} Different capital letters indicate a tendency ($P<0.1$); ^{a,b} Different lowercase letters indicate significant differences ($P<0.05$). ** Day effect $P<0.001$; ¤ Sex effect $P<0.05$; † Treatment effect $P<0.05$; ◊ Day*Sex*Treatment effect $P<0.05$.

Supplementary Table 4. Relative abundance (% of total reads) of bacterial families in female (♀) and male (♂) pigs receiving either the antibiotic (AB) or control (Control) treatment on days 0, 12, 28 and 97 of life.

	day 0					day 12				
	♀		♂			♀		♂		
	Control	AB	Control	AB	SEM	Control	AB	Control	AB	SEM
Enterobacteriaceae **	79.92	85.15	82.66	85.25	5.35	52.56 $^{B\ ab}$	65.46 $^{A\ a}$	44.09 $^{AB\ b}$	54.46 $^{AB\ ab}$	5.35
Clostridiaceae *	12.64	11.31	8.42	7.24	2.73	5.54	3.40	7.54	2.81	2.83
Ruminococcaceae **	0.67	0.25	0.52	0.10	2.71	12.85	10.61	14.05	14.00	2.80
Turicibacteraceae **	0.59	0.08	0.12	0.07	0.22	0.09	0.05	0.06	0.03	0.23
unclassified Clostridiales **	0.56	0.11	0.34	0.05	1.46	6.50 ^{A}	2.93 $^{B\ b}$	7.29 $^{AB\ a}$	7.39 $^{AB\ a}$	1.46
Campylobacteriaceae *	0.02	0	0.05	0	0.56	2.61 ^{a}	0.64 ^{b}	1.83 ^{ab}	0.77 ^{b}	0.56
BS_11 **	0.04	0.02	0.04	0	1.23	0.02	0	0.06	0	1.23
Christensenellaceae **	0.14	0.05	0.08	0.03	1.47	5.58 ^{a}	1.10 ^{b}	6.09 ^{a}	2.39 ^{ab}	1.47
Bifidobacteriaceae **	0.02	0	0	0.01	0.30	0.54 $^{B\ b}$	1.34 $^{A\ ab}$	1.44 $^{AB\ a}$	1.25 $^{AB\ ab}$	0.30
Coriobacteriaceae *	0.02	0.02	0.02	0	0.64	0.69 $^{AB\ b}$	1.48 $^{B\ ab}$	0.33 $^{AB\ b}$	3.06 $^{A\ a}$	0.64
unclassified Bacteroidales ** †	0.01	0	0.03	0	0.37	0.35	0.07	0.78	0.38	0.37
Lactobacillaceae ** †	0.07	0.03	0.06	0.01	0.21	0.10	0.14	0.36	0.20	0.21
Streptococcaceae ** ¤	0.14	0.06	0.10	0.03	0.06	0.02	0.01	0.01	0.01	0.06
Lachnospiraceae **	0.04	0.01	0.05	0.02	0.17	1.69 ^{a}	0.96 ^{b}	0.94 ^{b}	0.57 ^{b}	0.17
Veillonellaceae **	0.06	0.01	0.08	0.03	0.71	2.11	1.49	2.21	1.64	0.71
unclassified RF32 ** ¤ †	0	0	0.01	0	0.14	0	0	0	0	0.14
Desulfovibrionaceae **	0.01	0	0.01	0	0.17	1.61 ^{a}	0.60 ^{b}	0.89 ^{b}	0.98 ^{b}	0.17
Helicobacteriaceae *	0.09	0	0	0	0.40	0.10	0.20	0.11	0.02	0.40
Succinivibrionaceae ** ◊	0.01	0.01	0.09	0	1.35	0	0	0	0.01	1.35
Synergistaceae **	0.01	0	0	0.01	0.83	1.95	3.27	3.68	2.67	0.83

	day 28					day 97				
	♀		♂			♀		♂		
	Control	AB	Control	AB	SEM	Control	AB	Control	AB	SEM
Enterobacteriaceae **	0	0.46	2.02	0.27	7.60	1.39	0.75	1.69	0.74	7.60
Clostridiaceae *	6.18	8.85	10.83	4.67	3.89	12.94	13.10	10.85	15.40	3.89

Ruminococcaceae **	35.32	42.40	34.05	37.03	3.84	28.55	21.22	23.14	24.58	3.84
Turicibacteraceae **	1.60[a]	1.66[a]	1.69[a]	0.73[b]	0.31	0.04	0.05	0.05	0.07	0.31
unclassified Clostridiales **	12.96[ab]	12.44[ab]	9.58[b]	16.58[a]	2.04	15.08	12.45	13.03	12.47	2.04
Campylobacteriaceae *	0.47	0.97	1.01	0.52	0.80	1.72[b]	1.01[b]	1.40[b]	4.20[a]	0.80
BS_11 **	5.29[AB]	6.37[AB]	4.13[B]	8.94[A]	1.74	0.85[B ab]	0.14[AB b]	4.84[A a]	0.76[B ab]	1.74
Christensenellaceae **	6.43[b]	11.25[ab]	14.90[a]	13.03[a]	2.24	3.22	1.84	2.91	2.17	2.09
Bifidobacteriaceae **	0.03	0.03	0.01	0.01	0.43	0	0	0.02	0	0.43
Coriobacteriaceae *	0.54	0.28	0.28	0.31	0.91	0.47	0.42	0.43	0.45	0.91
unclassified Bacteroidales ** †	3.37[a]	0.92[b]	1.44[b]	1.24[b]	0.53	2.18	1.66	1.99	1.91	0.53
Lactobacillaceae ** †	0.28	0.28	0.36	0.35	0.29	2.72[a]	0.88[b]	2.30[a]	1.97[b]	0.29
Streptococcaceae ** ¤	0	0.05	0.01	0.02	0.09	1.15[a]	0.78[b]	0.62[b]	0.79[b]	0.09
Lachnospiraceae **	0.28	0.21	0.29	0.20	0.24	0.97	0.75	0.85	1.14	0.24
Veillonellaceae **	2.05	0.63	1.51	0.79	1.00	8.70[b]	14.45[a]	10.30[b]	10.31[b]	1.00
unclassified RF32 ** ¤ †	0.11	0.21	0.13	0.50	0.19	0.85[b]	1.18[b]	0.98[b]	2.04[a]	0.19
Desulfovibrionaceae **	1.53	1.10	1.34	1.30	0.24	0.49	0.37	0.42	0.43	0.24
Helicobacteriaceae *	2.84[a]	0.19[b]	1.08[b]	0.62[b]	0.54	0.36	0.23	0.25	0.35	0.54
Succinivibrionaceae ** ◊	1.02	0.23	0.51	2.15	1.92	9.40[b]	23.00[a]	17.30[a]	13.10[b]	1.92
Synergistaceae **	1.23	1.08	3.25	0.90	1.19	0	0.01	0.05	0	1.19

Data are presented as least-square means ± pooled standard error of the mean (SEM). A,B Different capital letters indicate a tendency (P<0.1); a,b Different lowercase letters indicate significant differences (P<0.05). ** Day effect P<0.001; * Day effect P<0.05; ¤ Sex effect P<0.05; † Treatment effect P<0.05; ◊ Day*Sex*Treatment effect P<0.05.

Supplementary Table 5. Relative abundance (% of total reads) of selected OTUs in female (♀) and male (♂) pigs receiving either the antibiotic (AB) or control (Control) treatment on days 0, 12, 28 and 97 of life.

		day 0					day 12				
		♀		♂			♀		♂		
		Control	AB	Control	AB	SEM	Control	AB	Control	AB	SEM
unclassified Enterobacteriaceae	OTU 1 **	71.46	77.84	76.28	78.07	4.90	47.26 $^{B\ ab}$	59.91 $^{A\ a}$	40.10 $^{AB\ b}$	49.74 $^{AB\ ab}$	5.07
Clostridium perfingens	OTU 2 **	4.74	7.41	5.16	4.24	1.72	3.45	1.82	4.51	1.39	1.72
unclassified Ruminococcaceae	OTU 3 ** † ◊	0	0.14	0.07	0.01	1.47	0.96	0.56	1.23	1.98	1.52
unclassified Enterobacteriaceae	OTU 4 **	3.02	3.36	3.15	3.24	0.21	1.88 ^{ab}	2.36 ^{a}	1.54 ^{b}	2.02 ^{ab}	0.21
unclassified Clostridiaceae	OTU 5 **	1.09	0.21	0.29	0.13	0.74	0.04	0.05	0.03	0	0.74
Succinivibrio	OTU 6 ** ◊	0	0.01	0.05	0	0.72	0	0	0	0.01	0.72
Succinivibrio	OTU 8 ** ◊	0	0	0.05	0	0.58	0	0	0	0	0.60
Ruminococcus	OTU 9 **	0	0.06	0	0	0.89	3.41	3.09	3.29	3.86	0.86
unclassified Christensenellaceae	OTU 11 **	0	0.02	0.04	0.01	0.55	1.46	1.57	1.13	0.57	0.57
unclassified Christensenellaceae	OTU 15 *	0	0.03	0	0	0.37	1.14	0.44	0.95	0.30	0.39
unclassified Clostridiales	OTU 18 *	0.06	0.03	0	0.01	0.41	0.73 $^{B\ ab}$	0.26 $^{AB\ b}$	1.64 $^{AB\ a}$	1.94 $^{A\ a}$	0.41
Campylobacter	OTU 19 **	0	0.01	0.03	0	0.64	2.30 ^{A}	2.06 ^{AB}	1.60 ^{AB}	0.62 ^{B}	0.64
unclassified Christensenellaceae	OTU 22 **	0	0.03	0.01	0.01	0.60	0.30	0.02	0.12	0	0.62
unclassified Clostridiales	OTU 23 *	0.02	0.02	0	0	0.38	0.66 ^{ab}	0.12 ^{b}	0.63 ^{ab}	1.27 ^{a}	0.40
unclassified Ruminococcaceae	OTU 24 **	0.05	0	0	0	0.25	0.03	0	0	0	0.25
Turicibacter	OTU 26 ** ¤	0.68	0.10	0.08	0.07	0.28	0.08	0.05	0.06	0.03	0.28
unclassified Bacteroidales	OTU 27 *	0	0.01	0.01	0	0.34	0.03	0.03	0.13	0.15	0.35
unclassified Ruminococcaceae	OTU 28 ** ¤	0	0.01	0	0	0.22	0.60	0.19	0.23	0.10	0.22
Anaerovibrio	OTU 35 ** ¤ † ◊	0	0	0.02	0	0.12	0	0	0	0	0.12
Lactobacillus	OTU 37 ** † ◊	0.04	0	0.02	0	0.17	0.01	0.04	0.05	0.05	0.17
Methanobrevibacter	OTU 40 ** †	0.04	0	0.02	0	0.20	0	0	0	0.01	0.21
unclassified Christensenellaceae	OTU 45 ** †	0	0.01	0	0	0.22	0.24	0.02	0.08	0	0.22
unclassified Ruminococcaceae	OTU 46 * ¤	0	0.01	0	0	0.22	0.15	0.02	0.18	0.18	0.22
Campylobacter	OTU 49 ** ¤ ◊	0	0.01	0.02	0	0.11	0	0	0.06	0.01	0.12
Anaerovibrio	OTU 51 ** ¤ † ◊	0	0	0.02	0	0.08	0	0	0	0	0.08
Desulfovibrio	OTU 52 ** ◊	0.02	0	0	0	0.07	0.75 $^{AB\ a}$	0.14 $^{B\ b}$	0.25 $^{AB\ b}$	0.33 $^{A\ b}$	0.08
unclassified Ruminococcaceae	OTU 56 **	0.09	0.04	0.01	0	0.10	0	0	0	0	0.10

Clostridium	OTU 57 **	0.32	0.01	0.02	0	0.16	0	0	0	0	0.16
unclassified Christensenellaceae	OTU 58 ** † ◊	0	0.01	0	0	0.13	0.08	0.14	0.09	0.01	0.14
unclassified Clostridiales	OTU 62 **	0	0.03	0.01	0	0.09	0	0	0	0	0.09
Ruminococcus	OTU 66 ** ¤	0	0.01	0.01	0	0.14	0	0	0.07	0	0.14
unclassified Clostridiales	OTU 71 ** ¤ † ◊	0.01	0	0.01	0	0.06	0	0	0	0	0.06
unclassified RF32	OTU 73 ** †	0	0	0.01	0	0.10	0	0	0	0	0.11
Streptococcus	OTU 76 ** ¤	0.09	0.02	0.04	0	0.06	0.01	0	0	0.01	0.06
unclassified Clostridiaceae	OTU 77 **	0.14	0.01	0.03	0.02	0.08	0	0.01	0	0	0.08
Helicobacter	OTU 81 ** †	0.09	0	0	0	0.14	0	0	0.02	0	0.15
unclassified Ruminococcaceae	OTU 85 **	0.02	0	0.01	0	0.05	0.01	0	0.01	0.01	0.05
Pyramidobacter piscolens	OTU 106 * † ◊	0	0	0.01	0	0.16	0.02	0.01	0.01	0.01	0.17
unclassified Ruminococcaceae	OTU 117 ** †	0	0	0	0	0.05	0.31 $^{AB\ a}$	0.09 $^{B\ b}$	0.20 $^{A\ ab}$	0.15 $^{AB\ b}$	0.05
Lactobacillus	OTU 118 ** †	0.01	0.01	0.02	0	0.05	0.02	0.01	0.02	0.02	0.05
Ruminococcus flavefaciens	OTU 124 *	0	0.01	0.01	0	0.14	0	0	0	0	0.15
Ruminococcus	OTU 174 ** ¤ † ◊	0	0	0	0	0.04	0	0	0	0	0.04

		day 28					day 97				
		♀		♂			♀		♂		
		Control	AB	Control	AB	SEM	Control	AB	Control	AB	SEM
unclassified Enterobacteriaceae	OTU 1 **	0.01	0.42	1.73	0.21	6.96	1.29	0.60	1.47	0.68	6.96
Clostridium perfingens	OTU 2 **	0	0.21	0.14	0.01	2.35	0.51	0	0.04	0.02	2.35
unclassified Ruminococcaceae	OTU 3 ** † ◊	11.83 ^{bc}	21.53 ^{a}	7.79 ^{bd}	14.95 ^{bc}	2.09	1.07	1.41	1.51	1.30	2.09
unclassified Enterobacteriaceae	OTU 4 **	0	0	0.05	0.02	0.29	0.05	0.05	0.08	0.02	0.29
unclassified Clostridiaceae	OTU 5 **	1.62 ^{AB}	2.89 ^{AB}	3.27 ^{A}	0.52 ^{B}	1.05	6.65	6.86	6.70	8.66	1.05
Succinivibrio	OTU 6 ** ◊	0.53	0.10	0.24	1.57	1.02	4.49 $^{AB\ b}$	11.37 $^{A\ a}$	8.77 $^{B\ a}$	6.48 $^{AB\ b}$	1.02
Succinivibrio	OTU 8 ** ◊	0.21	0.10	0.20	0.46	0.83	3.99 ^{c}	9.83 ^{a}	7.27 ^{b}	5.54 ^{bc}	0.83
Ruminococcus	OTU 9 **	0	0.31	0.43	0	1.18	0.08	0	0	0.02	1.18
unclassified Christensenellaceae	OTU 11 **	5.67 ^{a}	2.80 ^{b}	4.39 ^{ab}	4.04 ^{ab}	0.78	0	0	0	0.02	0.78
unclassified Christensenellaceae	OTU 15 *	0.82 $^{AB\ b}$	1.16 $^{AB\ b}$	2.67 $^{A\ a}$	1.39 $^{B\ ab}$	0.53	0.65	0.51	0.37	0.42	0.53
unclassified Clostridiales	OTU 18 *	1.78	0.67	0.97	1.13	0.59	0	0	0	0	0.59
Campylobacter	OTU 19 **	0.08	0.14	0.03	0.10	0.91	0.01	0	0.01	0	0.91
unclassified Christensenellaceae	OTU 22 **	4.11 $^{A\ a}$	0.77 $^{AB\ b}$	2.09 $^{B\ ab}$	2.59 $^{AB\ ab}$	0.85	0.26	0.18	0.27	0.12	0.85

unclassified Clostridiales	OTU 23 *	1.24 ^{ab}	2.10 ^{a}	0.46 ^{b}	1.08 ^{ab}	0.54	0.13	0.09	0.08	0.16	0.54
unclassified Ruminococcaceae	OTU 24 **	0.02	0.36	0.03	0.17	0.36	3.69 ^{a}	1.65 ^{b}	1.98 ^{b}	2.33 ^{b}	0.36
Turicibacter	OTU 26 ** ¤	1.50 ^{b}	2.86 ^{a}	1.60 ^{b}	0.71 ^{b}	0.40	0.05	0.05	0.06	0.07	0.40
unclassified Bacteroidales	OTU 27 *	2.63 ^{a}	0.25 ^{b}	0.68 ^{b}	0.46 ^{b}	0.48	1.01	0.83	1.05	0.87	0.48
unclassified Ruminococcaceae	OTU 28 ** ¤	1.98 ^{a}	1.75 ^{a}	1.51 ^{a}	0.53 ^{b}	0.32	0.09	0.09	0.10	0.05	0.32
Anaerovibrio	OTU 35 ** ¤ † ◊	0.27	0.02	0.06	0.05	0.18	0.91 ^{b}	3.12 ^{a}	1.42 ^{bc}	1.20 ^{bc}	0.18
Lactobacillus	OTU 37 ** † ◊	0.12	0.13	0.17	0.12	0.23	1.85 ^{a}	0.50 ^{b}	2.15 ^{a}	1.30 ^{ac}	0.23
Methanobrevibacter	OTU 40 ** †	0.91	0.91	0.89	0.28	0.29	1.55 ^{a}	0.32 ^{b}	0.74 ^{b}	0.52 ^{b}	0.29
unclassified Christensenellaceae	OTU 45 ** †	1.51 ^{a}	0.64 ^{b}	1.73 ^{a}	0.57 ^{b}	0.31	0.22	0.15	0.21	0.08	0.31
unclassified Ruminococcaceae	OTU 46 * ¤	0.40 $^{AB\ b}$	0.46 $^{B\ ab}$	1.28 $^{A\ a}$	1.39 $^{AB\ a}$	0.32	0.23	0.20	0.22	0.48	0.32
Campylobacter	OTU 49 ** ¤ ◊	0.01 $^{AB\ b}$	0.11 $^{B\ ab}$	0.50 $^{A\ a}$	0.01 $^{AB\ b}$	0.16	0.91 ^{b}	0.62 ^{b}	0.80 ^{b}	1.83 ^{a}	0.16
Anaerovibrio	OTU 51 ** ¤ † ◊	0.16	0.01	0.04	0.04	0.11	0.64 $^{B\ b}$	2.02 $^{AB\ a}$	0.95 $^{A\ b}$	0.79 $^{AB\ b}$	0.11
Desulfovibrio	OTU 52 ** ◊	0.40	0.42	0.43	0.37	0.11	0.13	0.04	0.03	0.06	0.11
unclassified Ruminococcaceae	OTU 56 **	0.06	0.37	0.30	0.28	0.14	1.30 ^{a}	0.67 ^{b}	0.60 ^{b}	0.76 ^{b}	0.14
Clostridium	OTU 57 **	0	0.03	0.11	0	0.22	0.92 $^{A\ ab}$	1.24 $^{AB\ a}$	0.30 $^{B\ b}$	1.31 $^{AB\ a}$	0.22
unclassified Christensenellaceae	OTU 58 ** † ◊	0.47 ^{b}	1.13 ^{a}	0.46 ^{b}	1.45 ^{a}	0.19	0.10	0.04	0.04	0.04	0.19
unclassified Clostridiales	OTU 62 **	0.23 $^{B\ b}$	0.92 $^{AB\ a}$	0.38 $^{AB\ b}$	0.56 $^{A\ b}$	0.12	0.63	0.50	0.50	0.45	0.12
Ruminococcus	OTU 66 ** ¤	0.08 ^{b}	0.18 ^{b}	0.27 ^{b}	0.85 ^{a}	0.20	0.50	0.45	0.49	0.88	0.20
unclassified Clostridiales	OTU 71 ** ¤ † ◊	0.01 ^{b}	0.26 ^{a}	0 ^{b}	0.01 ^{b}	0.09	0.66 ^{b}	1.19 ^{a}	0.62 ^{b}	0.76 ^{b}	0.09
unclassified RF32	OTU 73 ** †	0	0.09	0	0	0.14	0.51 ^{b}	1.09 ^{a}	0.56 ^{b}	1.19 ^{a}	0.14
Streptococcus	OTU 76 ** ¤	0	0	0	0	0.08	1.02 ^{a}	0.70 ^{b}	0.54 ^{b}	0.69 ^{b}	0.08
unclassified Clostridiaceae	OTU 77 **	0.08	0.03	0.19	0.21	0.11	0.83 ^{a}	0.54 ^{ab}	0.31 ^{b}	0.62 ^{a}	0.11
Helicobacter	OTU 81 ** †	0.98 ^{a}	0.11 ^{c}	0.70 ^{ab}	0.42 ^{bc}	0.20	0.19	0.14	0.14	0.18	0.20
unclassified Ruminococcaceae	OTU 85 **	0.17 ^{b}	0.44 ^{a}	0.23 ^{b}	0.57 ^{a}	0.07	0.39	0.32	0.42	0.29	0.07
Pyramidobacter piscolens	OTU 106 * † ◊	0.21 ^{b}	1.48 ^{a}	0.08 ^{b}	0.40 ^{b}	0.23	0	0	0	0	0.23
unclassified Ruminococcaceae	OTU 117 ** †	0.18 ^{ab}	0.06 ^{b}	0.29 ^{a}	0.15 ^{ab}	0.07	0.02	0	0	0	0.07
Lactobacillus	OTU 118 ** †	0.02	0.04	0.04	0.01	0.07	0.59 ^{a}	0.25 ^{b}	0.55 ^{a}	0.40 ^{b}	0.07
Ruminococcus flavefaciens	OTU 124 *	0.39 ^{ab}	0.15 ^{b}	1.04 ^{a}	0.04 ^{b}	0.20	0.07	0.10	0.12	0.04	0.20
Ruminococcus	OTU 174 ** ¤ † ◊	0	0	0	0	0.06	0.56 ^{a}	0.26 ^{b}	0.25 ^{b}	0.17 ^{b}	0.06

Data are presented as least-square means ± pooled standard error of the mean (SEM). ^{A,B} Different capital letters indicate a tendency (P<0.1); ^{a,b} Different lowercase letters indicate significant differences (P<0.05). ** Day effect P<0.001; * Day effect P<0.05; ¤ Sex effect P<0.05; † Treatment effect P<0.05;
◊ Day*Sex*Treatment effect P<0.05.
